# Effectiveness and acceptance of a web-based depression intervention during waiting time for outpatient psychotherapy: study protocol for a randomized controlled trial

**DOI:** 10.1186/s13063-018-2657-9

**Published:** 2018-05-22

**Authors:** Sasha-Denise Grünzig, Harald Baumeister, Jürgen Bengel, David Ebert, Lena Krämer

**Affiliations:** 1 Department of Rehabilitation Psychology and Psychotherapy, Institute of Psychology, Albert-Ludwigs-University Freiburg, Engelbergerstr. 41, 79085 Freiburg, Germany; 2 Department of Clinical Psychology and Psychotherapy, University of Ulm, Albert-Einstein-Allee 47, 89081 Ulm, Germany; 3 Department of Clinical Psychology and Psychotherapy, Institute of Psychology, Friedrich-Alexander University Erlangen-Nürnberg, Nägelsbachstr. 25a, 91052 Erlangen, Germany

**Keywords:** Web-based intervention, Internet, E-health, Waiting time, Waitlist, Depression, Outpatient treatment, Psychotherapy, Psychological health care

## Abstract

**Background:**

Due to limited resources, waiting periods for psychotherapy are often long and burdening for those in need of treatment and the health care system. In order to bridge the gap between initial contact and the beginning of psychotherapy, web-based interventions can be applied. The implementation of a web-based depression intervention during waiting periods has the potential to reduce depressive symptoms and enhance well-being in depressive individuals waiting for psychotherapy.

**Methods:**

In a two-arm randomized controlled trial, effectiveness and acceptance of a guided web-based intervention for depressive individuals on a waitlist for psychotherapy are evaluated. Participants are recruited in several German outpatient clinics. All those contacting the outpatient clinics with the wish to enter psychotherapy receive study information and a depression screening. Those adults (age ≥ 18) with depressive symptoms above cut-off (CES-D scale > 22) and internet access are randomized to either intervention condition (treatment as usual and immediate access to the web-based intervention) or waiting control condition (treatment as usual and delayed access to the web-based intervention). At three points of assessment (baseline, post-treatment, 3-months-follow-up) depressive symptoms and secondary outcomes, such as quality of life, attitudes towards psychotherapy and web-based interventions and adverse events are assessed. Additionally, participants’ acceptance of the web-based intervention is evaluated, using measures of intervention adherence and satisfaction.

**Discussion:**

This study investigates a relevant setting for the implementation of web-based interventions, potentially improving the provision of psychological health care. The results of this study contribute to the evaluation of innovative and resource-preserving health care models for outpatient psychological treatment.

**Trial registration:**

This trial has been registered on 13 February 2017 in the German clinical trials register (DRKS); registration number DRKS00010282.

**Electronic supplementary material:**

The online version of this article (10.1186/s13063-018-2657-9) contains supplementary material, which is available to authorized users.

## Background

Psychotherapy usually is a restricted resource, often associated with prolonged waiting periods for people seeking psychotherapeutic treatment. In Germany, on average, the waiting time to start psychotherapy is 4.5 months [1], with rural areas being particularly undersupplied [2]. These waiting periods are disadvantageous for people seeking help as well as the health care system. Individuals in need of mental health care experience an increased risk of chronification [3] and they are more dissatisfied with the help they receive [4]. They utilize more unspecific health care offers [5], causing high direct and indirect costs for the health care system [6]. Moreover, waiting periods contribute to people not starting psychotherapeutic treatment [7, 8] or not considering psychotherapy in the first place [9]. In order to address this issue, appropriate interventions should be offered to those unable to receive immediate psychotherapy.

One possibility to produce relief for people waiting for psychotherapy is the implementation of web-based self-help interventions. Web-based interventions have the potential to bridge treatment gaps [10, 11], as they can be applied flexibly, with comparably little time, space, and personnel resources [12, 13]. Since the late 1990s, a large body of research has emerged, confirming that web-based interventions are effective in reducing a range of psychological symptoms [14–18]. Particularly guided interventions offering feedback to participants have put forth promising effects [19]. Most recent research suggests that web-based interventions can be comparably effective to face-to-face psychotherapy [20]. While the efficacy of web-based interventions has been shown in various trials [21–24], the implementation of web-based interventions into health care systems worldwide is still in its infancy. One possibility to integrate web-based interventions into the health care system is their implementation during waiting periods. Offering web-based interventions to those waiting for psychotherapy may prove to be superior to mere waiting for several reasons: Individuals unable to enter psychotherapy could receive immediate access to an evidence-based intervention, therefore be provided with instant help; they could access the intervention 24 h a day, and use it in a familiar environment without time or travel costs [25]. For those in need of further treatment, interventions during waiting periods may facilitate progress in the subsequent face-to-face psychotherapy [26]. Hence, the implementation of a web-based intervention during waiting periods may prove to be particularly beneficial for both, help-seeking people and the adequate allocation of resources in our health care systems.

In order to assess the potential benefits of web-based interventions, they must be implemented in populations in need of help. One common complaint during waiting periods is depressive symptomatology [5]. With a point prevalence of about 8%, depressive symptoms are widespread in Germany [27], substantially reducing the quality of life of those affected [28, 29]. As almost half the patients in German outpatient clinics present with a depressive disorder [30, 31] and depressive symptoms often occur comorbid with other mental disorders [32], an intervention targeting the reduction of depressive symptoms is likely to be beneficial for many individuals waiting for psychotherapy.

The efficacy of web-based interventions for the reduction of depressive symptoms has been shown in several trials [22, 33]. However, there is limited evidence for their effectiveness and acceptance when implemented during waiting periods. To the best of our knowledge, three studies have examined web-based depression interventions during waiting periods [34–36]. Two studies reported intervention take-up rates between 26 and 53% [34, 37], high satisfaction among users [34] and large pre-post effect sizes of *d* = .75 [36]. The only randomized controlled trial testing a web-based depression intervention against mere waiting (treatment as usual) implemented an unguided intervention and had a number of methodological limitations, such as baseline assessment after randomization, substantial study dropout combined with missing intention-to-treat analyses and including participants without depressive symptoms [35]. At this point, no conclusions concerning the effectiveness of guided web-based depression interventions compared to treatment as usual during waiting periods can be drawn. Also, considering the results on intervention take-up and user satisfaction, findings concerning the acceptance of web-based interventions during waiting periods are quite heterogeneous and vary depending on the measure of acceptance (e.g., uptake vs. satisfaction rates) [34–36].

Taken together, the former studies highlight some of the challenges, such as low take-up rates [37], as well as the potentials of web-based interventions in this setting, such as high satisfaction among users [34] and possible reductions of depressive symptoms [36]. More research is needed to determine to what extent web-based interventions during waiting periods are superior to treatment as usual and which participant and intervention characteristics might impact on effectiveness and acceptance.

## Aims and research questions

The study aims at evaluating the effectiveness and acceptance of a guided web-based intervention for depressive individuals on a waitlist for outpatient psychotherapy. The following research questions are addressed:

- (Ia) Does the implementation of the web-based intervention have an effect on depressive symptoms when compared to waiting for psychotherapy without a web-based intervention? (effectiveness)
- (Ib) Does the implementation of the web-based intervention have an effect on other psychological symptoms, quality of life, and attitudes towards face-to-face psychotherapy and towards web-based interventions? (effectiveness)
- (II) How are intervention adherence (take-up rates, number of modules completed) and intervention satisfaction among depressive individuals waiting for face-to-face psychotherapy? (acceptance)
- (III) Which variables influence the effectiveness and acceptance of the web-based intervention (e.g., internet affinity, former psychotherapy)?

## Methods

### Design

**Fig. 1:**
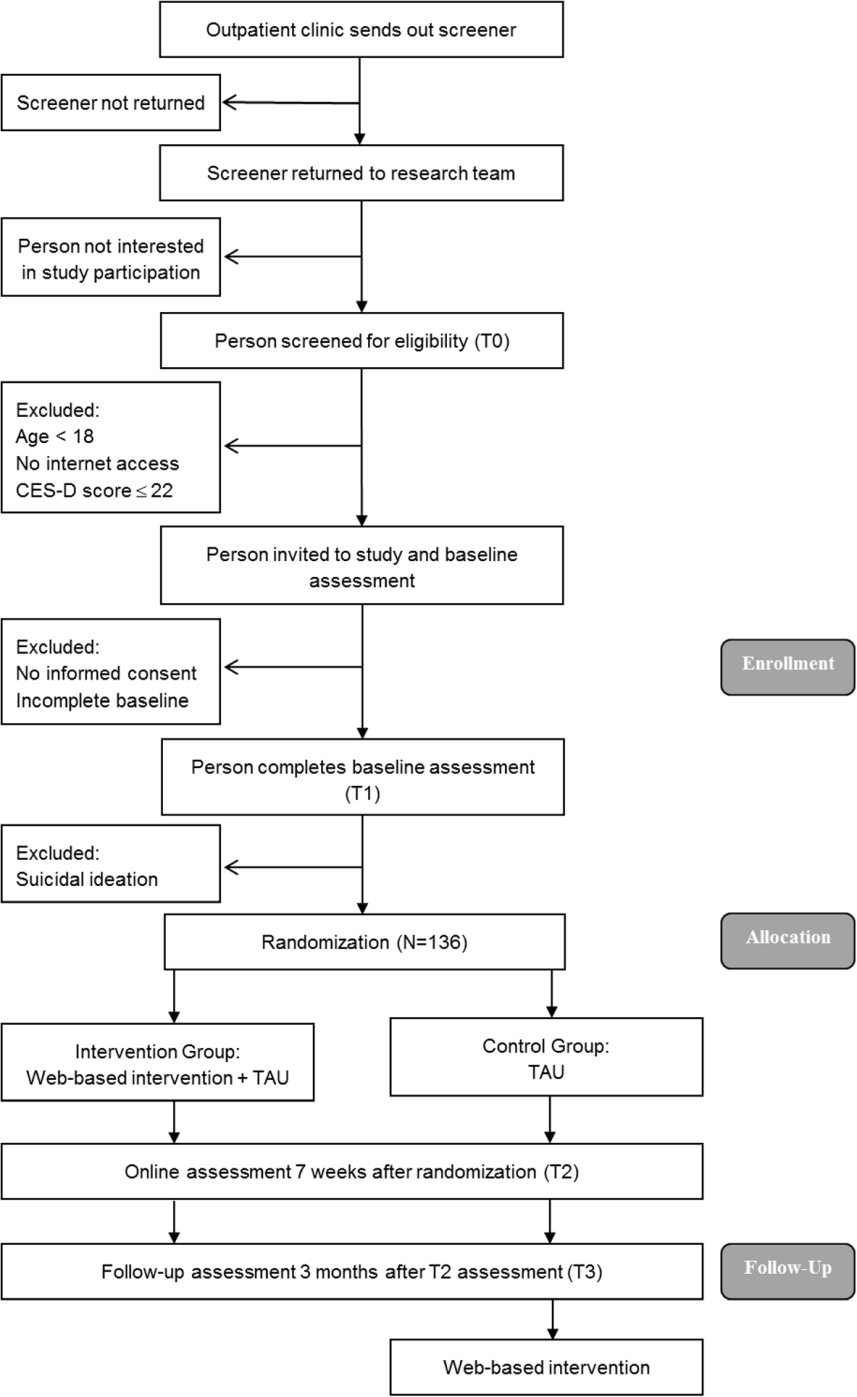
Study flow. *CES-D* Center for Epidemiologic Studies Depression Scale, *TAU* Treatment as usual

**Fig. 2:**
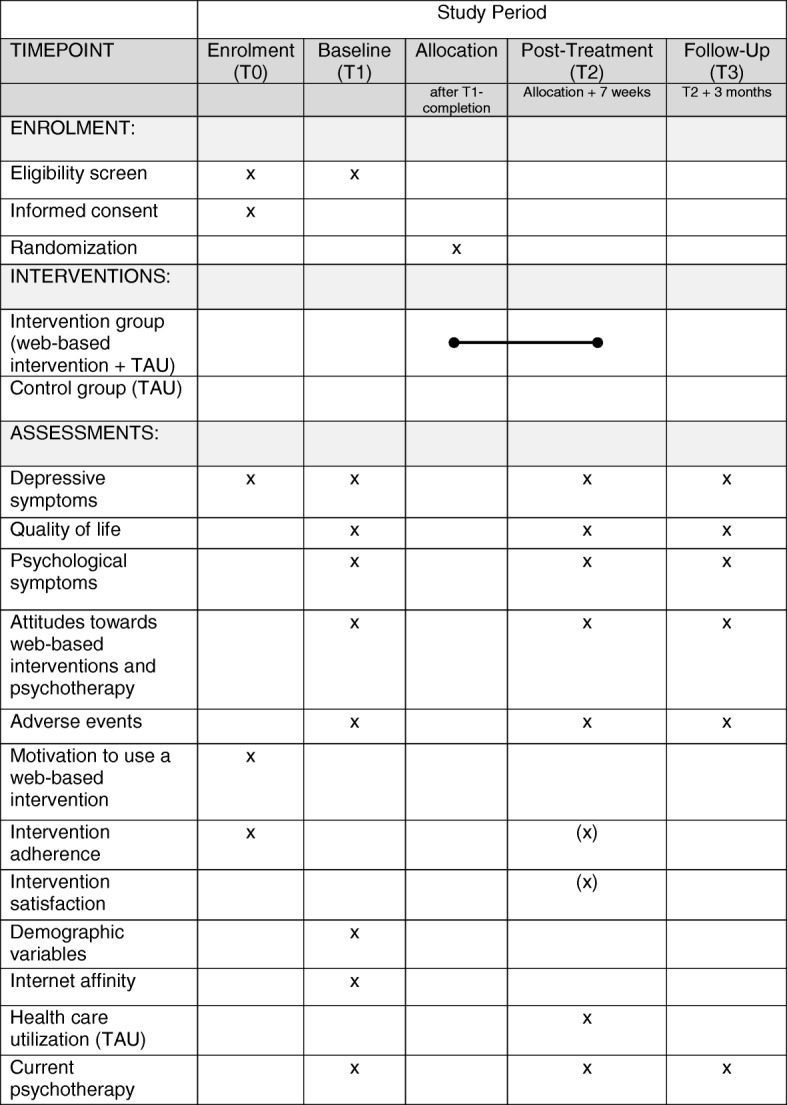
SPIRIT-figure

A two-arm randomized controlled trial (RCT) of parallel design is conducted in order to investigate the research questions. Participants are randomized either to the intervention group receiving immediate access to the web-based intervention, or the waiting control group receiving treatment as usual (TAU) until follow-up assessment, and subsequently access to the web-based intervention. TAU in the control condition means waiting for face-to-face psychotherapy while having access to all other services of health care (e.g., general practitioner). Assessments take place before randomization (T1), seven weeks after randomization (T2), and at 3-months follow-up (T3). Figure 1 displays the flow of participants following the SPIRIT guidelines [38]. Figure 2 and Additional file 1 are available online. All procedures have been approved by the ethics committee of the Albert-Ludwigs-University Freiburg (approval number 404/16). The trial is registered in the German clinical trials register under DRKS00010282. We conduct and report the trial in accordance with the CONSORT 2010 Statement [39], the supplement of the CONSORT statement for pragmatic effectiveness trials [40] and the guidelines for executing and reporting internet intervention research [41].

### Recruitment

Participants are recruited at several psychotherapeutic outpatient clinics in Germany. All cooperating outpatient clinics have a current wait of at least two months before individuals enter psychotherapy. Consecutively, all those accepted onto the waitlist of an outpatient clinic are informed about the ongoing study and receive study information and screening material. All individuals on the existing waitlists with a remaining waiting time of at least two months are also invited to fill in screening material. Screeners are sent back directly to the research team. Respondents are tested for eligibility to participate in the study (cf. inclusion criteria). Eligible respondents receive an e-mail providing further study information and a link to the digital informed consent sheet. All respondents giving their informed consent are enrolled in the online baseline assessment (T1) and then randomized either to the intervention group (IG) or the control group (CG). Participants of the IG are provided with a link to access the web-based intervention. Participants of the CG are given the opportunity to access the web-based intervention without guidance after follow-up assessment. Recruitment has started in February 2017 and is ongoing until both treatment arms have reached a size of *n* = 68. All procedures were specified prior to recruitment start and are conducted as explicated in the clinical registration form (DRKS00010282).

### Inclusion and exclusion criteria

Individuals waiting for psychotherapy in one of the cooperating outpatient clinics and interested in study participation are eligible when they indicate (1) an age of 18 years and over, (2) working internet access, and (3) depressive symptoms (CES-D score > 22; Center for Epidemiologic Studies Depression Scale [42]). Exclusion criteria are reduced to a minimum. This procedure allows for high external validity as it leads to a heterogeneous sample of people seeking outpatient psychotherapy independent of a particular diagnosis. It allows individuals with other primary diagnoses (e.g. anxiety disorder) to participate in the study, as long as they show substantial depressive symptoms. Comorbid psychological symptoms are detected with the Brief Symptom Inventory (BSI; [43]). As additional criterion, participants must (4) submit their informed consent, (5) complete baseline assessments and (6) state no suicidal ideation on the BSI item no. 9 (score < 2). In case of a BSI item score of 1, participants must agree to a non-suicide-contract before entering the study. In case of suicidal ideation throughout the study, we follow a firm suicide protocol, which has been approved by the ethics committee. Potential steps in case of suicidal ideation include information on further help, non-suicide contracts, and telephone calls by a licensed psychotherapist.

### Randomization and blinding

An independent researcher of the Methodological Support Centre of the Rehabilitation Research Network Freiburg, who is not elsewhere involved in the study, prepared randomization and allocation of participants in advance. As a means of randomization, an automated computer-based system is implemented (https://www.sealedenvelope.com/) using permuted block randomization with variable block sizes of 4, 6, and 8 (randomly arranged), in a ratio of 1:1. Randomization is stratified by outpatient clinic. The means for blinding in this study are limited. Still, data analysts will be blinded by creating syntaxes before adding the treatment condition variable to the data set.

### Proposed sample size

The sample size calculation is based on the difference in change in the primary outcome (depressive symptoms) from pre- to post-treatment in both treatment arms (intention-to-treat analysis). Considering recent meta-analytic effect sizes for web-based depression interventions, an effect size of *d* = 0.56 [33] can be expected and is considered feasible for the type of intervention (guided intervention, naturalistic study design, cf. [44]). On the basis of a two-sample t-test at a two-sided significance level of .05, the study is planned to detect this effect with 90% power. This requires a sample of 68 individuals in each arm.

With a target sample size of *N* = 136 for randomization, at least 597 screeners have to be sent out. Based on previous research, it is expected that about 50% of the contacted people are interested in study participation [37]. Further, about 65% of those interested are expected to score above the cut-off for depressive symptoms [45], resulting in 194 potential participants. A 30% loss is expected due to incomplete baseline, suicidal ideation or missing informed consent, leaving 136 participants for randomization.

### Intervention condition

The intervention in use (GET.ON Mood Enhancer) 46 consists of six consecutive modules, each about 30 min in length, and homework assignments. Participants are recommended to work on one or two modules per week. Adaptations of the intervention have proven effective in reducing depressive symptoms in varying samples [46–51]. The intervention has been slightly modified to suit the purpose of this setting (wording of the introduction, example participants, explanation of further treatment options, and text message support).

The intervention is based on behavioral activation [52] and problem-solving therapy [53]. The core elements of the intervention are (1) psycho-education, (2) behavioral activation, (3) systematic problem solving, and (4) optional lections on sleep, rumination, and relaxation. Additionally, participants receive access to an electronic mood diary. Participants receive a semi-standardized feedback after each completed module by an e-coach (trained psychologist) in order to enhance adherence. Feedback includes positive reinforcement of the participants’ assignments and encouragement to continue working with the intervention. E-coaches are not otherwise involved in the study. Additionally, participants can chose to activate text message support (42 text messages, one per day). The text messages are standardized and remind the participants of their weekly assignments and repeat specific lessons. Text message prompts and guidance have been shown to be very beneficial for the efficacy and adherence to web-based interventions [33, 54, 55]. Participants also receive standardized e-mails reminding them of unfinished tasks. Participants can access the intervention at any time and from all web-enabled devices. Each module closes with a short questionnaire, assessing subjective usefulness of the module, the location and time spent working on the module, and the level of concentration.

Participants’ access to TAU (e.g., visits to other medical practitioners) is not restricted. A detailed description of TAU is obtained at post-treatment. Any contact between the research team and study participants (e.g., online assessments, e-mail reminders) is standardized for all study participants (IG and CG) and reduced to a minimum.

### Control condition

Participants of the waiting control group are informed that they can access the web-based intervention after follow-up assessments. Again, access to TAU until follow-up assessment is not restricted. As for the intervention group, a detailed description of TAU is obtained at post-treatment.

## Outcome measures

Table 1 gives an overview of assessments at screening, baseline, post-treatment and follow-up.

**Table 1:** Overview of measurements

Variables	Instrument	Screening	T1	T2	T3
Inclusion/exclusion criteria					
Age	SR	x			
Internet access	SR	x			
Depressive symptoms	CES-D	x			
Non-Suicidality	BSI item 9		x		
Effectiveness					
Primary outcome					
Depressive symptoms	CES-D	x	x	x	x
Secondary outcomes					
Depressive symptoms (categorical)	PHQ-9		x	x	x
Health-related quality of life	SF-12		x	x	x
Psychological symptoms	BSI		x	x	x
Attitudes towards psychotherapy	ATSPPH-SF		x	x	x
Attitudes towards web-based interventions	ATSPPH-SF (adapted version)		x	x	x
Adverse events	INEP			x	
Acceptance					
Motivation to use a web-based intervention	SR	x			
Intervention adherence	Response and dropout rate	x		(x)	
Intervention satisfaction	CSQ-8			(x)	
Sample characteristics					
Demographic variables	SR		x		
Internet affinity	IAS		x		
Health care utilization	FIMA			x	
Current Psychotherapy	SR		x	x	x

Note. *T1* baseline, *T2* post-treatment, *T3* follow-up, *x* intervention and control group, *(x)* only intervention group, *SR* self-report, *CES-D* Center for Epidemiologic Studies Depression Scale, *BSI* Brief Symptom Inventory, *PHQ-9* Patient Health Questionnaire, *SF-12* Health Survey, *ATSPPH-SF* Attitude Towards Seeking Professional Psychological Help- Short Form, *INEP* Inventory for the Assessment of Negative Effects of Psychotherapy, *CSQ-8* Client Satisfaction Questionnaire, *IAS* Internet Affinity Scale, *FIMA* Questionnaire for Health-Related Resource Use in an Elderly Population

### Effectiveness measures

#### Primary outcome

##### Depressive symptoms

Depressive symptoms are assessed using the Center for Epidemiologic Studies Depression Scale (CES-D; German version [42]). The CES-D consists of 20 items measuring the global level of depressive severity within the last week on a 4-point Likert scale. The total score ranges from 0 to 60, with higher scores indicating more severe depressive symptoms. Its internal consistency of *α* = .92 in clinical samples is very good [42].

#### Secondary outcomes

##### Depressive symptoms

The Patient Health Questionnaire (PHQ-9; [56]) allows a categorical classification of depression severity, distinguishing between moderate, moderately severe and severe major depression. It consists of nine items and assesses depressive symptoms of the past two weeks. Its internal consistency reaches values of *α* = .88 [57].

##### Health related quality of life

To assess health related quality of life, the SF-12 Health Survey [58] is used. The instrument provides two subscales, measuring physical and mental quality of life components. It consists of 12 items, rated on scales between two and five points. Reliability and validity of the SF-12 have been well documented (*α* = .77; [58]).

##### Psychological symptoms

The Brief Symptom Inventory (BSI; [43]) is applied to measure psychological symptoms of the past week. Its 53 items are rated on a 5-point Likert scale and cover symptoms of somatization, obsessive compulsion, interpersonal sensitivity, depression, anxiety, hostility, phobic anxiety, paranoid ideation and psychoticism. The Global Severity Index (GSI) reflects the respondents’ overall level of psychological distress. Validity and reliability of the BSI are well-established (*α* = .91; [43]). Additionally, item number 9 of the BSI serves as an indicator for suicidal ideation.

##### Attitudes towards psychotherapy

Participants’ attitudes towards face-to-face psychotherapy are measured by the Attitude Towards Seeking Professional Psychological Help Scale - Short Form (ATSPPH-SF; [59]). This instrument consists of 10 items that are rated on a 4-point Likert scale. The psychometric properties of the questionnaire are good (*α* = .78; [60]).

##### Attitudes towards web-based interventions

An adapted version of the Attitude Towards Seeking Professional Psychological Help Scale - Short Form (ATSPPH-SF; [59]) is applied to assess the attitude towards web-based interventions. Compared to the original version of the ATSPPH-SF, solely the term “psychotherapy” is replaced by the term “online training” in each item. The number of items remains unchanged.

##### Adverse events

The Inventory for the Assessment of Negative Effects of Psychotherapy (INEP; [61]) consists of 21 items dealing with potential side effects of psychotherapy. In accordance with previous studies [62–65], the instrument is adapted for the particular setting of web-based interventions, resulting in the deletion of 6 face-to-face specific items. The remaining 15 items are scored on a 4-point Likert scale. The reliability of this instrument is good (*α* = .86; [61]).

#### Acceptance measures

##### Intervention adherence

The adherence to the intervention in use is depicted by the take-up rate at the level of recruitment and the number of modules completed. Additionally, the intervention dropout relates to the number of intervention completers (≥ 5 modules; [46]) and non-completers in the IG. For a better understanding of intervention dropouts, all non-completers are asked to indicate reasons for their non-completion.

##### Intervention satisfaction

The Client Satisfaction Questionnaire (CSQ-8; [66]) measures client satisfaction with health care services. Following Boss and colleagues [67], we use an adapted version for the evaluation of satisfaction with web-based interventions for IG-participants at post-treatment. The scale consists of eight items, rated on a 4-point Likert scale. The adapted scale has been validated, indicating high reliability and construct validity [67]. In addition to intervention satisfaction, one item assesses technical difficulties dealing with the intervention. This item will be evaluated separately.

##### Motivation to use a web-based intervention

In order to gain an understanding of how interested respondents differ from non-interested respondents at the level of recruitment, the screening questionnaire includes five items dealing with respondents’ motivation to try a web-based interventions. Three items assess respondents’ anticipated usefulness of a web-based intervention, one item assesses their willingness to try a web-based intervention, and one item assesses respondents’ computer skills with regards to the application of a web-based intervention. All items are rated on a 4-point Likert scale.

#### Sample characteristics

##### Demographic variables

Socio-demographic variables are assessed at baseline, based on the recommendations of Deck and Röckelein [68]. These variables include age, gender, family status, education, employment, diseases, and former psychotherapy.

##### Internet affinity

Internet affinity is measured using the Internet Affinity Scale (IAS; [69]). The IAS measures internet affinity and frequency of internet usage with six items to be rated on a 5-point Likert scale. The scale’s reliability is good (*α* = .84; [69]). One additional item assesses computer-related competencies and will be evaluated separately.

##### Health care utilization

For the assessment of utilized health care services, the Questionnaire for Health-Related Resource Use in an Elderly Population (FIMA; [70]) is applied in an adapted version; items dealing with seniority-specific aspects, such as the usage of nursing services or domestic help, are left out. The remaining ten items assess the number of utilized health care services of the past eight weeks, as well as the current intake of medication. One additional item assesses the use of additional psychological health care options, such as bibliotherapy and self-help groups.

##### Current Psychotherapy

At all points of assessment participants indicate their current motivation and their perceived need for psychotherapy, and whether they are currently receiving face-to-face psychotherapy. Participants indicating their current receipt of psychotherapy are asked how many sessions they have had and when their first appointment has been. Participants not receiving face-to-face psychotherapy are asked to indicate reasons.

## Statistical analyses

### Effectiveness analyses

Analyses will be based on an intention-to-treat principle by including all randomized participants into the analyses. Primary and secondary outcomes will be analysed using a linear mixed model, assuming data are missing at random. The mixed model for the primary outcome (depression) will include group, time (all three points of assessment) and the interaction of group and time as fixed effects and recruiting outpatient clinic as random effect. Secondary outcomes will be analysed accordingly. We will calculate between-group effect sizes for the primary outcome using the post-treatment depression means and their pooled observed standard deviation. Additional per protocol analyses will include only those participants who have not started psychotherapy until the end of all study procedures, completing all three assessments, and, regarding the intervention group, at least five intervention modules.

We will evaluate the clinical relevance of any given development in a generalized linear model by estimating numbers of treatment response and deterioration (based on the reliable change index; [71]) and symptom remission (ADS-L score < 22; [41]), and by calculating the number needed to treat (NNT) for one more remitted participant.

Potential moderators influencing treatment effects will be analysed in the mixed model analysis. As there is at this point little research concerning moderating variables in the field [72], these analyses follow an exploratory approach. Potential influencing variables include socio-demographics, internet affinity or attitudes towards psychotherapy.

Analyses will be performed using an alpha level of .05 and two-sided tests. All analyses will be conducted using IBM SPSS.

### Acceptance analyses

Intervention adherence will be calculated by assessing the response rate of returned screeners with interest in study participation and the number of modules completed by intervention group participants. Recruitment and dropout rates will be examined using absolute and percentage frequencies. Participants’ satisfaction with the intervention (T2) will be reported descriptively. Potential predictors influencing intervention adherence and intervention satisfaction (e.g., age, depressive symptoms, internet affinity, former psychotherapy) will be assessed in an exploratory multiple regression analysis.

## Discussion

This study investigates the effectiveness and acceptance of a guided web-based intervention for individuals with depressive symptoms seeking psychotherapy. Participants are individuals with elevated depressive symptoms on a waitlist for psychotherapy at several psychotherapeutic outpatient clinics, who consent to applying an intervention for mood improvement. A randomized controlled trial is conducted, comparing an intervention group receiving immediate access to a web-based intervention to a waiting control group. We expect the intervention group to benefit from the web-based intervention with regard to depressive symptoms, psychological symptoms and quality of life at post intervention and 3-months follow up. In an exploratory approach, the acceptance of the web-based intervention during waiting periods will be assessed, taking various sources of information into account (e.g., take-up and dropout rates).

This study features a number of strengths. While a series of trials have highlighted the efficacy of web-based depression interventions [22], more research on their effectiveness in practical settings is needed [73]. This study focuses on the applicability of a web-based intervention in a setting where low-intensity interventions are scarce and urgently needed. At the same time the waiting period differs from other investigated settings, as participants are distinctly seeking face-to-face psychotherapy. Thus, participants might perceive the web-based intervention as less credible, thinking that the “real treatment” is yet to come, potentially reducing the effectiveness of the intervention in this setting [74, 75]. The study is based on a solid methodology, applying a randomized controlled trial with three times of assessment. The pragmatic study design allows high external validity. As inclusion and exclusion criteria are reduced to a minimum, the investigated sample consists of individuals with depressive symptoms seeking outpatient psychotherapy. In line with current standards, analyses will be based on an intention-to-treat principle and performed using linear mixed-model analyses.

This study also has a few limitations which deserve note. As the study will be conducted in the context of routine mental health care, the waiting periods of participants will vary depending on the capacities of the cooperating outpatient clinics. Some participants will presumably start psychotherapy between post- and follow-up assessments. However, due to the limited time span between post and follow-up assessments, the take-up of a subsequent psychotherapy cannot be reliably assessed. Per protocol analyses will be conducted including only those participants who have not started psychotherapy until the completion of follow-up assessments. Due to the routine care setting and in favor of external validity, we include participants who may suffer from other primary psychological symptoms (e.g. anxiety). These participants might benefit more from other disorder-specific interventions. Nonetheless, trials have implemented web-based depression interventions in routine care settings with high external validity and put forth large effect sizes (e.g. [36, 37]). Similar effects are expected in this study as all participants suffer from depressive symptoms and take an informed choice to partake in an intervention for mood improvement.

When it comes to web-based interventions, considerable dropout rates have been reported [76]. The participants dropping out of the intervention will be asked for their reasons to do so, thus dropouts will be used for a deeper understanding of intervention acceptance and applicability. In an attempt to minimize intervention dropouts, we implement a web-based intervention with guidance [19]. Additionally, per protocol analysis will be performed for a high quality data analysis. Another limitation is that the main outcome data is based on self-reports. This is a frequently conducted approach with a favorable study cost–validity balance, still clinician-rated outcome measures would be beneficial. We have selected measures with high internal consistency and validity. In line with other research in the field (e.g., [63, 77]), retest-reliabilities are not reported as studies vary greatly in terms of evaluation times and sample characteristics, which makes retest-reliabilities difficult to interpret in the context of this study design. Also, due to related effort, no analyses of cost-effectiveness are performed in this study. Last, the recruitment of participants has started in February 2017; however, the study has been registered before the beginning of recruitment and no changes have been made to the registry.

The results of this study will be of great relevance for daily clinical practice, as they reflect the applicability of an evidence-based self-help treatment option for individuals seeking psychotherapeutic treatment. Addressing the problem of prolonged waiting periods, it is essential to investigate which treatment options are effective and accepted by those in need. Evidence-based interventions with minimal effort for outpatient clinics are scarce and urgently needed. Thus, implementing a web-based intervention in this setting may be beneficial for those on a waitlist and health care providers. Also, this study investigates a model to viably integrate web-based interventions into the health care system. Considering the growing interest and realization of new treatment approaches, such as stepped-care models [78, 79], web-based interventions have the potential to play an important role as low-intensity interventions in the treatment of depression [80]. As Kazdin and Blase [81] emphasize, innovative treatment approaches are needed to decrease the burden of mental diseases on a large scale.

## Trial Status

This is the first protocol version. Recruitment has started in February 2017 and will presumably be completed in summer 2018.

## Additional file

Additional file 1: SPIRIT-checklist. (DOC 162 kb)

## Abbreviations

*ATSPPH-SF*: Attitude Towards Seeking Professional Psychological Help Scale-Short
*BSI*: Brief Symptom Inventory
*CES-D*: Center for Epidemiologic Studies Depression Scale
*CG*: Control group
*CSQ*: Client Satisfaction Questionnaire
*FIMA*: Questionnaire for Health-Related Resource Use in an Elderly Population
*GSI*: Global Severity Index
*IAS*: Internet Affinity Scale
*IG*: Intervention group
*INEP*: Inventory for the Assessment of Negative Effects of Psychotherapy
*PHQ*: Patient Health Questionnaire
*RCT*: Randomized controlled trial
*TAU*: Treatment as usual
