# Correction to: Effectiveness and acceptance of a web-based depression intervention during waiting time for outpatient psychotherapy: study protocol for a randomized controlled trial

**DOI:** 10.1186/s13063-018-2806-1

**Published:** 2018-07-19

**Authors:** Sasha-Denise Grünzig, Harald Baumeister, Jürgen Bengel, David Ebert, Lena Krämer

**Affiliations:** 1 Department of Rehabilitation Psychology and Psychotherapy, Institute of Psychology, Albert-Ludwigs-University Freiburg, Engelbergerstr. 41, 79085 Freiburg, Germany; 2 Department of Clinical Psychology and Psychotherapy, University of Ulm, Albert-Einstein-Allee 47, 89081 Ulm, Germany; 3 Department of Clinical Psychology and Psychotherapy, Institute of Psychology, Friedrich-Alexander University Erlangen-Nürnberg, Nägelsbachstr. 25a, 91052 Erlangen, Germany

## Correction

Following publication of the original article [1], the author reported the errors in the figure and in text reference in the published article. These errors are detailed below. The original article has been corrected.

1. Figure 2 SPIRIT-figure appears in the manuscript although it is only meant as online supplementary material.

2. In the Design section, the citation for the supplementary online materials should be “Additionally, Fig. 2 (SPIRIT-figure) and File 1 (SPIRIT-checklist) are available online” instead of “Fig. 2 and Additional file 1 are available online”.
