# Supplementary material for: Effectiveness and acceptance of a web-based depression intervention during waiting time for outpatient psychotherapy: study protocol for a randomized controlled trial
Source: Trials. 2018 May 22;19:285. doi: 10.1186/s13063-018-2657-9 (PMC5964713; doi:10.1186/s13063-018-2657-9)
Supplement: Supplementary file 1 — SPIRIT-checklist. (DOC 162 kb) [file 13063_2018_2657_MOESM1_ESM.doc]

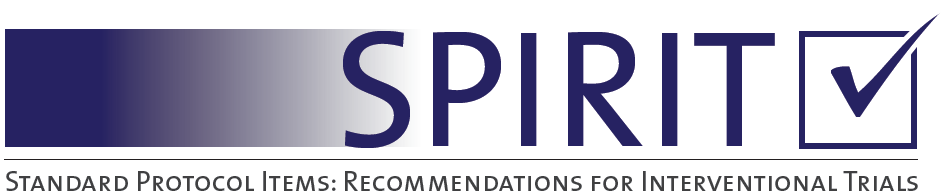


SPIRIT 2013 Checklist: Recommended items to address in a clinical trial protocol and related documents*

| Section/item | ItemNo | Description |
| --- | --- | --- |
| **Administrative information** | | |
| Title | 1 | Effectiveness and acceptance of a web-based depression intervention during waiting time for outpatient psychotherapy: study protocol for a randomized controlled trial |
| Trial registration | 2a | German clinical trials register: DRKS00010282 |
|  | 2b | see table below |
| Protocol version | 3 | 1^st^ protocol version, February 9^th^ 2018 |
| Funding | 4 | This study is funded by the Young Researchers Academy “Health Services Research” Baden-Württemberg (Nachwuchsakademie Versorgungsforschung Baden-Württemberg) and the Cooperative Doctoral Study Course "Health Services Research: Collaborative Care" (Kooperatives Promotionskolleg Versorgungsforschung Collaborative Care), which in turn are brought forward by the Ministry of Science, Research and the Arts Baden-Württemberg. |
| Roles and responsibilities | 5a | Sasha-Denise Grünzig, M.Sc. Psych. (corresponding author)  Department of Rehabilitation Psychology and Psychotherapy  Institute of Psychology  Albert-Ludwigs-University Freiburg  Engelbergerstr. 41, 79085  Freiburg, Germany  [sashi.gruenzig@psychologie.uni-freiburg.de](mailto:sashi.gruenzig@psychologie.uni-freiburg.de)  Tel: +49 761 203-3044  Prof. Dr. Harald Baumeister  Department of Clinical Psychology and Psychotherapy  University of Ulm  Albert-Einstein-Allee 47, 89081  Ulm, Germany  harald.baumeister@uni-ulm.de  Tel: +49 731-50-32800  Prof. Dr. Dr. Jürgen Bengel  Department of Rehabilitation Psychology and Psychotherapy  Institute of Psychology  Albert-Ludwigs-University Freiburg  Engelbergerstr. 41, 79085  Freiburg, Germany  bengel@psychologie.uni-freiburg.de  Tel: +49 761-203-3046  Dr. David Ebert  Department of Clinical Psychology and Psychotherapy  Institute of Psychology  Friedrich-Alexander University Erlangen-Nürnberg  Nägelsbachstr. 25a, 91052  Erlangen, Germany  David.ebert@fau.de  Tel: +49 9131-85-67566  Dr. Lena Krämer  Department of Rehabilitation Psychology and Psychotherapy  Institute of Psychology  Albert-Ludwigs-University Freiburg  Engelbergerstr. 41, 79085  Freiburg, Germany  kraemer@psychologie.uni-freiburg.de  Tel: +49 761-203-9315  LK conceived of the study and initiated the study design. JB and SG contributed to the design of this study. SG executes recruitment and data acquisition, she wrote the draft of the study protocol. LK and JB supervise the implementation. HB and DE constitute the study advisory board. HB, JB, DE, and LK revised the draft of the manuscript, read and approved the final manuscript. |
|  | 5b | Dr. Lena Krämer  Department of Rehabilitation Psychology and Psychotherapy  Institute of Psychology  Albert-Ludwigs-University Freiburg  Engelbergerstr. 41, 79085  Freiburg, Germany  kraemer@psychologie.uni-freiburg.de  Tel: +49 761-203-9315 |
|  | 5c | The funding source had no role in the design of this study and will not have any role during its execution, analyses, interpretation of the data, or decision to submit results. |
|  | 5d | SG is responsible for recruitment, data acquisition and data analyses. LK and JB are supervisors of the trial. HB and DE constitute the study advisory board. The cooperating outpatient clinics inform potential participants about the ongoing trial. The Methodological Support Centre of the Rehabilitation Research Network Freiburg is responsible for randomization. |
| Introduction |  |  |
| Background and rationale | 6a | Psychotherapy usually is a restricted resource, often associated with prolonged waiting periods for people seeking psychotherapeutic treatment. In Germany, on average, the waiting time to start psychotherapy is 4.5 months (Bundespsychotherapeutenkammer, 2011), with rural areas being particularly undersupplied (Apolinário-Hagen & Tasseit, 2015). These waiting periods are disadvantageous for people seeking help as well as the health care system (Siponen & Välimäki, 2003; Helbig & Hoyer, 2007). One possibility to produce relief for people waiting for psychotherapy is the implementation of web-based self-help interventions. Web-based interventions have the potential to bridge treatment gaps (Musiat, Goldstone & Tarrier, 2014), as they can be applied flexibly, with comparably little time, space, and personnel resources (Eells et al., 2014). Since the late 1990s, a large body of research has emerged, confirming that web-based interventions are effective in reducing depressive symptoms (Richards & Richardson, 2012; Königbauer et al., 2017; Ebert et al., 2015). This study aims at evaluating the effectiveness and acceptance of a guided web-based intervention for depressive individuals on a waitlist for outpatient psychotherapy. |
|  | 6b | The control condition receives the treatment that is usually provided during waiting periods (TAU) in order to investigate the superiority of the web-based intervention compared to standard routine care in this setting. |
| Objectives | 7 | (Ia) Does the implementation of the web-based intervention have an effect on depressive symptoms when compared to waiting for psychotherapy without a web-based intervention? (effectiveness)  (Ib) Does the implementation of the web-based intervention have an effect on other psychological symptoms, quality of life, and attitudes towards face-to-face psychotherapy and towards web-based interventions? (effectiveness)  (II) How are intervention adherence (take-up rates, number of modules completed) and intervention satisfaction among depressive individuals waiting for face-to-face psychotherapy? (acceptance)  (III) Which variables influence the effectiveness and acceptance of the web-based intervention (e.g., internet affinity, former psychotherapy)? |
| Trial design | 8 | This is a two-arm randomised controlled superiority trial of parallel design with a 1:1 allocation ratio with depressive symptomatology as primary outcome. |
| Methods: Participants, interventions, and outcomes | | |
| Study setting | 9 | Participants are recruited at several psychotherapeutic outpatient clinics in Germany. A list of participating outpatient clinics can be obtained from the corresponding author. |
| Eligibility criteria | 10 | All cooperating outpatient clinics have a current wait of at least two months before individuals enter psychotherapy.  Individuals waiting for psychotherapy in one of the cooperating outpatient clinics and interested in study participation are eligible when they indicate (1) an age of 18 years and over, (2) working internet access, and (3) depressive symptoms (CES-D score > 22; Center for Epidemiologic Studies Depression Scale). Exclusion criteria are reduced to a minimum. This procedure allows for high external validity as it leads to a heterogeneous sample of people seeking outpatient psychotherapy independent of a particular diagnosis. It allows individuals with other primary diagnoses (e.g. anxiety disorder) to participate in the study, as long as they show substantial depressive symptoms. Comorbid psychological symptoms are detected with the Brief Symptom Inventory (BSI). As additional criterion, participants must (4) submit their informed consent, (5) complete baseline assessments and (6) state no suicidal ideation on the BSI item no. 9 (score < 2). In case of a BSI item score of 1, participants must agree to a non-suicide-contract before entering the study. In case of suicidal ideation throughout the study, we follow a firm suicide protocol which has been approved by the ethics committee. |
| Interventions | 11a | Eligible participants are randomised to either intervention or control condition. Participants of the intervention group receive an e-mail providing a link to access the web-based intervention.  The intervention in use (GET.ON Mood Enhancer) consists of six consecutive modules, each about 30 minutes in length, and homework assignments. Participants are recommended to work on one or two modules per week. The intervention is based on behavioral activation (Lewinsohn, 1974) and problem-solving therapy (D’Zurilla & Nezu, 1986). The core elements of the intervention are (1) psycho-education, (2) behavioral activation, (3) systematic problem solving, and (4) optional lections on sleep, rumination, and relaxation. Additionally, participants receive access to an electronic mood diary. Participants receive a semi-standardized feedback after each completed module by an e-coach (trained psychologist) in order to enhance adherence. Feedback includes positive reinforcement of the participants’ assignments and encouragement to continue working with the intervention. E-coaches are not otherwise involved in the study. Additionally, participants can chose to activate text message support (42 text messages, one per day). The text messages are standardized and remind the participants of their weekly assignments and repeat specific lessons. Participants also receive standardized e-mails reminding them of unfinished tasks. Participants can access the intervention at any time and from all web-enabled devices. Each module closes with a short questionnaire, assessing subjective usefulness of the module, the location and time spent working on the module, and the level of concentration. |
|  | 11b | In line with our ethical approval, we assess potential adverse events at post-treatment (T2) and, in case of frequent reports of adverse events, we will abort the running trial. Trial participants are closely monitored. In case of suicidal ideation, they are treated according to a firm protocol excluding highly suicidal participants from the study. |
|  | 11c | Intervention group participants receive semi-standardized written feedback by a psychologist and optional standardized text messages to enhance intervention adherence. If participants do not continue the intervention for ten days, they receive a standardized e-mail reminding them of the next module. The research team monitors the number of completed modules. |
|  | 11d | Participants’ access to TAU (e.g., visits to other medical practitioners) is not restricted. A detailed description of TAU is obtained at post-treatment (T2). |
| Outcomes | 12 | Primary outcome is the change in depressive symptoms compared between the two conditions across the three points of assessment. Statistically significant differences between the two groups will be interpreted in terms of effect sizes. Additionally, the clinical relevance of any given development will be analysed (cf. Statistical methods). Secondary outcomes for intervention effectiveness include changes in quality of life, psychological symptoms, and attitudes towards web-based interventions and psychotherapy.  Intervention acceptance will be analysed using response and dropout rates as well as participants’ satisfaction with the intervention. |
| Participant timeline | 13 | Cf. Figure 1 of the study protocol (Study Flow) |
| Sample size | 14 | The sample size calculation is based on the difference in change in the primary outcome (depressive symptoms) from pre- to post-treatment in both treatment arms (intention-to-treat analysis). Considering recent meta-analytic effect sizes for web-based depression interventions, an effect size of d = 0.56 (Richard & Richardson, 2012) can be expected and is considered feasible for the type of intervention (guided intervention, naturalistic study design, cf. Gellatly et al., 2007). On the basis of a two-sample t-test at a two-sided significance level of .05, the study is planned to detect this effect with 90% power. This requires a sample of 68 individuals in each arm.  With a target sample size of N = 136 for randomization, at least 597 screeners have to be sent out. Based on previous research, it is expected that about 50% of the contacted people are interested in study participation (Kenter et al., 2013). Further, about 65% of those interested are expected to score above the cut-off for depressive symptoms (Krämer et al., 2014), resulting in 194 potential participants. A 30% loss is expected due to incomplete baseline, suicidal ideation or missing informed consent, leaving 136 participants for randomization. |
| Recruitment | 15 | All cooperating outpatient clinics will continue to send out study information to persons on their waitlists until the target sample size is reached. |
| **Methods: Assignment of interventions (for controlled trials)** | | |
| Allocation: |  |  |
| Sequence generation | 16a | An independent researcher of the Methodological Support Centre of the Rehabilitation Research Network Freiburg, who is not elsewhere involved in the study, prepared randomization and allocation of participants in advance. As a means of randomization, an automated computer-based system is implemented (https://www.sealedenvelope.com/) using permuted block randomization with variable block sizes of 4, 6, and 8 (randomly arranged), in a ratio of 1:1. Randomization is stratified by outpatient clinic. |
| Allocation concealment mechanism | 16b | Allocation concealment is ensured as participants are not randomised until they have been included into the trial, which is after all baseline measurements have been completed. |
| Implementation | 16c | The research team enrols new participants and reports to the independent researcher form the Methodological Support Centre of the Rehabilitation Research Network Freiburg. He then conducts randomisation for each new participant and reports back to the research team. The research team then assigns participants to the respective condition. |
| Blinding (masking) | 17a | The means for blinding in this study are limited. Still, data analysts will be blinded by creating syntaxes before adding the treatment condition variable to the data set. |
| **Methods: Data collection, management, and analysis** | | |
| Data collection methods | 18a | All outcome measure are collected via online questionnaires.  Depressive symptoms are assessed at all points of assessment using the Center for Epidemiologic Studies Depression Scale (CES-D; German version; Hautzinger et al., 2012). The CES-D consists of 20 items measuring the global level of depressive severity within the last week on a 4-point Likert scale. The total score ranges from 0-60, with higher scores indicating more severe depressive symptoms. Its internal consistency of α = .92 in clinical samples is very good (Hautzinger et al., 2012).  Additionally, the Patient Health Questionnaire (PHQ-9; Kroenke & Spitzer, 2002) allows a categorical classification of depression severity, distinguishing between moderate, moderately severe and severe major depression. It is applied at T1, T2 and T3. It consists of nine items and assesses depressive symptoms of the past two weeks. Its internal consistency reaches values of α = .88 (Löwe et al., 2004).  To assess health related quality of life, the SF-12 Health Survey (Ware, Kosinski & Keller, 1996) is used at T1, T2 and T3. The instrument provides two subscales, measuring physical and mental quality of life components. It consists of 12 items, rated on scales between two and five points. Reliability and validity of the SF-12 have been well documented (α = .77; Ware, Kosinski & Keller, 1996).  The Brief Symptom Inventory (BSI; Franke, 2000) is applied at T1, T2 and T3 to measure psychological symptoms of the past week. Its 53 items are rated on a 5-point Likert scale and cover symptoms of somatization, obsessive compulsion, interpersonal sensitivity, depression, anxiety, hostility, phobic anxiety, paranoid ideation and psychoticism. The Global Severity Index (GSI) reflects the respondents’ overall level of psychological distress. Validity and reliability of the BSI are well-established (α = .91; Franke, 2000). Additionally, item number 9 of the BSI serves as an indicator for suicidal ideation.  Participants’ attitudes towards face-to-face psychotherapy are measured at T1, T2 and T3 by the Attitude Towards Seeking Professional Psychological Help Scale - Short Form (ATSPPH-SF; Fischer & Farina, 1995). This instrument consists of 10 items that are rated on a 4-point Likert scale. The psychometric properties of the questionnaire are good (α = .78; Elhai, Schweinle & Anderson, 2008).  An adapted version of the Attitude Towards Seeking Professional Psychological Help Scale - Short Form (ATSPPH-SF; Fischer & Farina, 1995) is applied at T1, T2 and T3 to assess the attitude towards web-based interventions. Compared to the original version of the ATSPPH-SF, solely the term “psychotherapy” is replaced by the term “online training” in each item. The number of items remains unchanged.  The Inventory for the Assessment of Negative Effects of Psychotherapy (INEP; Ladwig, Rief & Nestoriuc, 2014) is applied at T2 and consists of 21 items dealing with potential side effects of psychotherapy. In accordance with previous studies, the instrument is adapted for the particular setting of web-based interventions, resulting in the deletion of 6 face-to-face specific items. The remaining 15 items are scored on a 4-point Likert scale. The reliability of this instrument is good (α = .86; Ladwig, Rief & Nestoriuc, 2014). |
|  |  | The adherence to the intervention in use is depicted by the take-up rate at the level of recruitment and the number of modules completed. Additionally, the intervention dropout relates to the number of intervention completers (≥ 5 modules; Buntrock et al., 2015) and non-completers in the IG. For a better understanding of intervention dropouts, all non-completers are asked to indicate reasons for their non-completion.  The Client Satisfaction Questionnaire (CSQ-8; Nguyen, Attkisson & Stegner, 1983) measures client satisfaction with health care services. Following Boss and colleagues (Boss et al., 2016), we use an adapted version for the evaluation of satisfaction with web-based interventions for IG-participants at post-treatment. The scale consists of eight items, rated on a 4-point Likert scale. The adapted scale has been validated, indicating high reliability and construct validity (Boss et al., 2016). In addition to intervention satisfaction, one item assesses technical difficulties dealing with the intervention. This item will be evaluated separately.  In order to gain an understanding of how interested respondents differ from non-interested respondents at the level of recruitment, the screening questionnaire includes five items dealing with respondents’ motivation to try a web-based interventions. Three items assess respondents’ anticipated usefulness of a web-based intervention, one item assesses their willingness to try a web-based intervention, and one item assesses respondents’ computer skills with regards to the application of a web-based intervention. All items are rated on a 4-point Likert scale.  Socio-demographic variables are assessed at baseline, based on the recommendations of Deck and Röckelein (Deck & Röckelein, 1999). These variables include age, gender, family status, education, employment, diseases, and former psychotherapy.  Internet affinity is measured at baseline using the Internet Affinity Scale (IAS; Papacharissi & Rubin, 2000). The IAS measures internet affinity and frequency of internet usage with six items to be rated on a 5-point Likert scale. The scale’s reliability is good (α = .84; Papacharissi & Rubin, 2000). One additional item assesses computer-related competencies and will be evaluated separately.  For the assessment of utilized health care services at T2, the Questionnaire for Health-Related Resource Use in an Elderly Population (FIMA; Seidl et al., 2015) is applied in an adapted version; items dealing with seniority-specific aspects, such as the usage of nursing services or domestic help, are left out. The remaining ten items assess the number of utilized health care services of the past eight weeks, as well as the current intake of medication. One additional item assesses the use of additional psychological health care options, such as bibliotherapy and self-help groups.  At all points of assessment participants indicate their current motivation and their perceived need for psychotherapy, and whether they are currently receiving face-to-face psychotherapy. Participants indicating their current receipt of psychotherapy are asked how many sessions they have had and when their first appointment has been. Participants not receiving face-to-face psychotherapy are asked to indicate reasons. |
|  | 18b | Participants not completing online assessments will be sent a reminder via e-mail and, if necessary given a phone call to encourage the completion of all assessments. |
| Data management | 19 | All data will be handled and stored as described in our data protection plan and ethical approval. All data will be entered and checked by SG. A proportion of the data will be double checked by another researcher. |
| Statistical methods | 20a | Analyses will be based on an intention-to-treat principle by including all randomized participants into the analyses. Primary and secondary outcomes will be analysed using a linear mixed model, assuming data are missing at random. The mixed model for the primary outcome (depression) will include group, time (all three points of assessment) and the interaction of group and time as fixed effects and recruiting outpatient clinic as random effect. Secondary outcomes will be analysed accordingly. We will calculate between-group effect sizes for the primary outcome using the post-treatment depression means and their pooled observed standard deviation. Additional per protocol analyses will include only those participants who have not started psychotherapy until the end of all study procedures, completing all three assessments, and, regarding the intervention group, at least five intervention modules.  We will evaluate the clinical relevance of any given development in a generalized linear model by estimating numbers of treatment response and deterioration (based on the reliable change index; Jacobson & Truax, 1991) and symptom remission (ADS-L score < 22), and by calculating the number needed to treat (NNT) for one more remitted participant.  Potential moderators influencing treatment effects will be analysed in the mixed model analysis. As there is at this point little research concerning moderating variables in the field, these analyses follow an exploratory approach. Potential influencing variables include socio-demographics, internet affinity or attitudes towards psychotherapy.  Analyses will be performed using an alpha level of .05 and two-sided tests. All analyses will be conducted using IBM SPSS.  Intervention adherence will be calculated by assessing the response rate of returned screeners with interest in study participation and the number of modules completed by intervention group participants. Recruitment and dropout rates will be examined using absolute and percentage frequencies. Participants’ satisfaction with the intervention (T2) will be reported descriptively. Potential predictors influencing intervention adherence and intervention satisfaction (e.g., age, depressive symptoms, internet affinity, former psychotherapy) will be assessed in an exploratory multiple regression analysis. |
| **Methods: Monitoring** | | |
| Data monitoring | 21a | Data is constantly monitored by the trial investigator (corresponding author) in order to reveal any adverse events or technical problems. |
|  | 21b | Data collection will continue until the targeted sample size is reached. In case of frequent reports of adverse events, we will abort the running trial in line with our ethical approval. |
| Harms | 22 | Adverse events are assessed at post-treatment (cf. data collection). In case of frequent reports of adverse events, we will abort the running trial in line with our ethical approval. In case of suicidal ideation, we follow a firm suicide protocol, which has been approved by the ethics committee of the University of Freiburg. Adverse events will be reported in the outcome paper. |
| Auditing | 23 | n.a. |
| Ethics and dissemination | | |
| Research ethics approval | 24 | All procedures have been approved by the ethics committee of the Albert-Ludwigs-University Freiburg (approval number 404/16). |
| Protocol amendments | 25 | Any amendments of the study protocol will be reported in future publications of this trial. |
| Consent or assent | 26a | Interested persons send in their screening material including a signed consent to receive further study information. The trial investigator sends an e-mail to interested persons fulfilling the inclusion criteria. The e-mail provides further study information and a link to the digital informed consent sheet. Interested persons submit their informed consent online. The trial investigator receives the informed consent sheets. |
|  | 26b | n.a. |
| Confidentiality | 27 | All data will be handled and stored as described in our data protection plan and ethical approval. All personal data will be destroyed after study completion. All questionnaire data will be destroyed after 10 years. |
| Declaration of interests | 28 | The authors declare that they have no competing interests. |
| Access to data | 29 | Only the project team in Freiburg has access to all collected data. |
| Ancillary and post-trial care | 30 | All participants are on the waitlist for outpatient psychotherapy. Hence, they receive the treatment as usual provided by their outpatient clinic and presumably psychotherapy. |
| Dissemination policy | 31a | The results of this trial will be published in scientific journals and presented at research conferences. Every attempt will be made to reduce to an absolute minimum the interval between the completion of data collection and the release of the study results. |
|  | 31b | The authorship of future publications of this trial has been documented in advance. Contributions of each author will be described transparently. No professional writers will be hired. |
|  | 31c | The study protocol is published in an open access format. Participant-level datasets and statistical codes will not be published. |
| Appendices |  |  |
| Informed consent materials | 32 | All participant materials have been approved by the ethics committee and can be obtained (in German) from the corresponding author. |
| Biological specimens | 33 | n.a. |

| World Health Organization Trial Registration Data Set | |
| --- | --- |
| Primary registry and trial identifying number: | German clinical trials register: DRKS00010282 |
| Date of registration in primary registry: | February 13th 2017 |
| Secondary identifying numbers: | n.a. |
| Source(s) of monetary or material support: | This study is funded by the Young Researchers Academy “Health Services Research” Baden-Württemberg (Nachwuchsakademie Versorgungsforschung Baden-Württemberg) and the Cooperative Doctoral Study Course "Health Services Research: Collaborative Care" (Kooperatives Promotionskolleg Versorgungsforschung Collaborative Care), which in turn are brought forward by the Ministry of Science, Research and the Arts Baden-Württemberg. |
| Primary and secondary sponsors: | Cf. sources of monetary or material support |
| Contact for public queries: | Sasha-Denise Grünzig, M.Sc. Psych.  Department of Rehabilitation Psychology and Psychotherapy  Institute of Psychology  Albert-Ludwigs-University Freiburg  Engelbergerstr. 41, 79085  Freiburg, Germany  [sashi.gruenzig@psychologie.uni-freiburg.de](mailto:sashi.gruenzig@psychologie.uni-freiburg.de)  Tel: +49 761 203-3044 |
| Contact for scientific queries: | Sasha-Denise Grünzig, M.Sc. Psych.  Department of Rehabilitation Psychology and Psychotherapy  Institute of Psychology  Albert-Ludwigs-University Freiburg  Engelbergerstr. 41, 79085  Freiburg, Germany  [sashi.gruenzig@psychologie.uni-freiburg.de](mailto:sashi.gruenzig@psychologie.uni-freiburg.de)  Tel: +49 761 203-3044 |
| Public title | Effectiveness and acceptance of a web-based depression intervention during waiting time for outpatient psychotherapy: study protocol for a randomized controlled trial |
| Scientific title | Effectiveness and acceptance of a web-based depression intervention during waiting time for outpatient psychotherapy: study protocol for a randomized controlled trial |
| Countries of recruitment: | Germany |
| Health condition(s) or problem(s) studied: | Depressive symptoms |
| Intervention(s): | Web-based intervention for the reduction of depressive symptoms vers. TAU-control condition |
| Key inclusion and exclusion criteria: | Individuals waiting for psychotherapy in one of the cooperating outpatient clinics and interested in study participation are eligible when they indicate (1) an age of 18 years and over, (2) working internet access, and (3) depressive symptoms (CES-D score > 22; Center for Epidemiologic Studies Depression Scale). As additional criterion, participants must (4) submit their informed consent, (5) complete baseline assessments and (6) state no suicidal ideation on the BSI item no. 9 (score < 2). In case of a BSI item score of 1, participants must agree to a non-suicide-contract before entering the study. In case of suicidal ideation throughout the study, we follow a firm suicide protocol which has been approved by the ethics |
| Study type | This is a two-arm randomised controlled superiority trial of parallel design with a 1:1 allocation ratio with depressive symptomatology as primary outcome |
| Date of first enrolment | February 2017 |
| Target sample size | 136 |
| Recruitment status | Recruiting |
| Primary outcome(s) | Change of depressive symptoms |
| Key secondary outcomes | Acceptance of the intervention; changes in quality of life and psychological symptoms |
